# Does caffeine reduce postoperative bowel paralysis after elective laparoscopic colectomy? (CaCo trial): study protocol for a randomized controlled trial

**DOI:** 10.1186/s13063-016-1297-1

**Published:** 2016-04-04

**Authors:** Christina Kruse, Sascha A. Müller, René Warschkow, Cornelia Lüthi, Walter Brunner, Lukas Marti, Michael Christian Sulz, Bruno M. Schmied, Ignazio Tarantino, Ulrich Beutner

**Affiliations:** Department of General, Visceral, Endocrine and Transplantation Surgery, Kantonsspital St. Gallen, Rorschacher Str. 95, 9007 St. Gallen, Switzerland; Department of General, Visceral, Endocrine and Transplantation Surgery, Kantonsspital St. Gallen - Spital Rorschach, Heidenerstrasse 11, 9400 Rorschach, Switzerland; Division of Gastroenterology and Hepatology, Kantonsspital St. Gallen, Rorschacher Str. 95, 9007 St. Gallen, Switzerland; Department of General, Visceral and Transplantation Surgery, University of Heidelberg, Im Neuenheimer Feld 110, 69120 Heidelberg, Germany

**Keywords:** Colon surgery, Postoperative bowel paralysis, Caffeine, Postoperative management, Laparoscopic colectomy, Ileus

## Abstract

**Background:**

Postoperative bowel paralysis is common after abdominal operations, including colectomy. As a result, hospitalization may be prolonged, thereby leading to increased cost. A recent randomized controlled trial showed that the consumption of regular black coffee after colectomy is associated with a significantly faster resumption of intestinal motility. The mechanism by which coffee stimulates intestinal motility is unknown, but caffeine seems to be the most likely stimulating agent. Thus, the effect of caffeine on postoperative bowel activity after colon surgery will be analyzed in this trial, herein referred to as CaCo.

**Methods/design:**

Patients scheduled for elective laparoscopic colectomy or upper rectum resection are eligible to participate in this double-blinded, placebo-controlled, randomized trial. Patients fulfilling all inclusion criteria will be allocated after the surgical procedure to one of three treatment arms: 100 mg caffeine, 200 mg caffeine, or placebo (corn starch). Patients will take the capsules containing the study medication three times daily with a meal. The primary endpoint of the study is the time to a solid bowel movement. The study treatment will be stopped after the patient produces a solid bowel movement or has taken ten capsules, whichever occurs first. To determine the colonic passage time, patients will take a capsule with radiopaque markers at breakfast for the first 3 days after surgery. On the fourth day, the location of the markers will be determined with an abdominal X-ray scan. Further secondary objectives are the postoperative morbidity and mortality, well-being, sleeping behavior, and length of hospital stay.

The study size was calculated to be 180 patients with an interim analysis occurring after 60 patients.

**Discussion:**

From a previous study investigating coffee, evidence exists that caffeine might have a positive influence on the postoperative bowel activity. This double-blinded, placebo-controlled, randomized trial tries to show that caffeine will shorten the postoperative bowel paralysis and, thus, will improve recovery and shorten the hospital stay after colon surgery.

**Trial registration:**

Clinicaltrials.gov NCT02510911

Swiss National Clinical Trials Portal SNCTP000001131

**Electronic supplementary material:**

The online version of this article (doi:10.1186/s13063-016-1297-1) contains supplementary material, which is available to authorized users.

## Background

Postoperative bowel paralysis is common after abdominal operations, including colectomy [1]. In most patients, after colon resection, normal intestinal function returns slowly. Surgical stress, pain, and bowel paralysis contribute to this delay. A delay in bowel function (ileus) may lead to a prolonged hospital stay, hospital-acquired infections or complications, and pulmonary compromise [1]. Patients with postoperative bowel paralysis have symptoms of pain, distention, and emesis. They may require treatments such as nasogastric tube, decompression, analgesia, fluid, and electrolyte replacement. Consequentially, hospitalization may be prolonged, thereby leading to increased costs.

Because of the significant implications of bowel paralysis after colectomy, surgeons have developed various strategies to minimize postoperative bowel paralysis.

Multimodal approaches to treat postoperative bowel paralysis in colorectal surgery include early feeding; the restricted use of nasogastric tubes; thoracic epidural analgesia; nonspecific pharmacologic agents, such as water-soluble contrast agents (gastrografin); specific agents, such as alvimopan; and a selective μ-receptor opioid antagonist [1–5].

Chewing gum is another approach to stimulate bowel function after surgery [6]. Asao et al. found an earlier return of bowel function and a trend toward earlier hospital discharge in patients who chewed gum after laparoscopic colectomy. Gum is postulated to activate the cephalic-vagal reflex, which is usually enhanced by food, and to increase the production of the gastrointestinal hormones associated with bowel motility [6].

Coffee is a popular beverage with well-known effects on general well-being, the central nervous system, and the cardiovascular system [7], but information is limited regarding its effects on gastrointestinal function. The gastrointestinal system can be stimulated by food with a substantial caloric content, acidity, osmolality, or volume load. Brewed and filtered coffee is hypotonic, as is water, which serves as a control in most studies investigating coffee. The hypotonicity of coffee, therefore, is unlikely to be responsible for the effects on the gastrointestinal tract. More likely, the ingredients of coffee have a pharmacological effect on the gut.

Twenty-nine percent of a group of volunteers who answered a questionnaire concerning their bowel habits claimed that coffee induced a desire to defecate [8]. Rectosigmoid motor responses to coffee were investigated with a multiport manometry in these volunteers who claimed that coffee caused a desire to defecate and in volunteers who did not so claim. An increase in colonic motor activity was found after 4 min of ingestion of both regular and decaffeinated coffee in the responders but not in the nonresponders. After water, no response in any volunteer was found. Rao et al. performed an ambulatory manometry with a catheter positioned from the rectum up to the midtransverse colon [9]. They studied the effects of 240 ml of regular coffee, decaffeinated coffee, water, and a 1000-kcal meal on colonic motility. Regular coffee, decaffeinated coffee, and the meal induced more colonic activity and more propagated contractions than water. Caffeinated coffee was concluded to stimulate colonic motor activity at a magnitude comparable to a high-calorie meal, 60 % stronger than water, and 25 % stronger than decaffeinated coffee.

A recently performed randomized controlled trial at the University of Heidelberg showed that the consumption of regular black coffee after colectomy is safe and is associated with a significantly faster resumption of intestinal motility [10]. Neither previous studies nor the study from Heidelberg could clearly explain the mechanisms by which coffee stimulates intestinal motility. The most obvious stimulating agent would be caffeine. Therefore, we would like to investigate the role of caffeine on bowel motor activity in this study.

This study is restricted to laparoscopic colectomies because colectomies are increasingly performed by this access technique (our institution: 50–60 % of all colectomies in 2014, German speaking countries: 25–30 %). Since the time to bowel movement is significantly longer after open surgery, open and laparoscopic approaches cannot be compared without significant bias.

## Methods/design

### Study objectives

With this study, we would like to address whether caffeine has a beneficial effect on bowel paralysis after colon resections and whether the side effects of caffeine are within a tolerable range. If we can prove our assumption, caffeine would be a very cost-effective drug to treat postoperative bowel paralysis and could help to shorten the length of the hospitalization.

### Study design

This trial is a double-blinded, placebo-controlled, randomized trial with three arms. Patients will be randomized after the surgical procedure to receive either 3 × 100 mg caffeine, 3 × 200 mg caffeine, or 3 × 250 mg corn starch (placebo) daily. Study participants will be equally allocated to the three arms (1:1:1). The study is an investigator-initiated trial, and the principal investigator also acts as the sponsor. The study will be performed at two sites (St. Gallen and Rorschach) of a major, nonacademic, Swiss medical center. Both sites belong to the same department of surgery.

The study protocol was drafted following the SPIRIT statement (see Additional file 1 for a detailed SPIRIT checklist).

### Outcomes

The primary outcome of the study will be the return to normal bowel function after colon resection. The primary endpoint will be the time to the first bowel movement after surgery. Secondary objectives are the safety of the caffeine treatment, particularly the degree and perception of the known effects of caffeine, such as agitation and insomnia, and the recovery from surgery, morbidity, and mortality.

### Study population

Study participants will be recruited from patients of the Department of General, Visceral, Endocrine and Transplantation Surgery scheduled for laparoscopic elective colon and upper rectum resection. The indication for surgery (oncologic or nononcologic) is not a selection criterion.

In particular, the patient has to fulfill the following criteria to be considered for study inclusion:

- The patient is scheduled for elective laparoscopic colectomy (right or left hemicolectomy, segmental resection, extended hemicolectomy, sigmoid resection, or upper rectum (anastomosis higher than 7 cm above the anal verge)).
- The patient’s age is equal to or over 18 years old. As long as the patient is fit for surgery, no upper age limit exists.
- The patient gives informed consent before surgery.

The following criteria will exclude the patients from trial participation:

- Participation in another concurrent interventional trial
- Need for a stoma (colostomy or ileostomy) or reversal of a stoma, if the patient had complete bowel obstruction
- Known hypersensitivity or allergy to caffeine/coffee
- Expected lack of compliance (patient is notorious for not following medical instructions, like taking medications as prescribed, or following necessary diets)
- American Society of Anesthesiologists (ASA) Physical Status Score of IV or V
- Impaired mental state or language problems
- Alcoholism or drug abuse
- Previous extensive abdominal surgery (any open or laparoscopic abdominal surgery except laparoscopic appendectomy, cholecystectomy, or hernia repair)
- Inflammatory bowel disease
- Clinically significant cardiac arrhythmia (patient suffers from physical complaints or is on regular medication)
- Cardiac insufficiency (like the previous criterion as safe guard against the effects of caffeine)
- Pregnancy, lactation, or childbearing potential without using adequate contraception
- Intake of opioid analgesics, or steroids > 5 mg/day for ≥ 7 days before surgery
- Under antidepressive medication
- Liver cirrhosis or compromised liver function (MELD score > 15) (Caffeine is metabolized in liver cells, and impaired liver function would result in a prolonged half-life of the caffeine.)

The final decision to include (i.e., to randomize) the patient in the trial will be made at the end of the surgery. The following criteria have to be fulfilled:

- No major change of the planned surgery (like conversion to open, unplanned placement of a stoma, additional resections of adjacent organs, or other major unplanned procedures)
- No epidural anesthesia used during or after surgery

### Study withdrawal and stopping medication

After randomization, the patients can withdraw at any time if they own wish without giving any reason.

Study treatment will be stopped for the following reasons:

- Any adverse event resulting from surgery that might interfere with the study drug or might lead to meaningless endpoint data (e.g., organ failure, anastomotic failure, or postoperative dementia). Due to the complexities of postoperative morbidity, no list of fixed criteria will be established. If such an event occurs before the first study medication will be taken, the patient will be withdrawn from the study and replaced by another patient.
- Serious adverse events occur that are definitively or probably related to the study drug.
- Tachycardia, defined as a pulse of over 120/min three times within 1 hour, occurs.
- In the investigator’s opinion, continuation of the treatment is detrimental to the patient’s well-being.

Unless the patient wishes to withdraw from the study, all scheduled examinations will be performed as planned as long as they are meaningful and do not pose an unnecessary burden to the patient (e.g., no X-ray examination will be performed if treatment is stopped very early or if the patient has multiple vomiting events during the first 3 days postoperatively). Thus, stopping the medication does not automatically constitute withdrawal from the study.

The investigator can withdraw a study patient if a reoperation before the return to normal bowel function is required, an epidural analgesia was placed, if there are signs of renal insufficiency or an acute renal insufficiency, or if the patient clearly is not compliant (repeated refusal to take study medication or is consuming caffeine-containing beverages or food).

### Endpoints

The primary endpoint is the time to the first bowel movement. The time will be measured from the time of wound closure to the patient’s first bowel movement. Patients were advised before study participation to remember or note their first stool after surgery. A study nurse will inquire daily whether bowel movement has occurred.

The definitions of the secondary endpoints are indicated as follows. The time to tolerance of solid food is measured from the end of surgery until the patient tolerates the intake of solid food. Tolerance of food is defined as the first time the patient is able to eat solid food (any food requiring chewing) without vomiting or experiencing significant nausea within 4 h after the meal and without reversion to only enteral fluids.

Time to first flatus is the time from the end of surgery until the patient’s first flatus. The passage of flatus will be determined by questioning the patient; the passage of a bowel movement will be determined by referring to the nursing records or by the clinical judgment of the investigator or designee following questioning of the patient.

Postoperative vomiting events, determined from the nursing records, refer to the number of times the patient has to vomit.

Colonic passage time will be determined using ten radiopaque markers (Colon Transit Radiopaque Markers, P & A Mauch, 4142 Münchenstein, Switzerland, Ref: CTT6V10, CE1253). From day 1 to day 3 after surgery, the patients will receive one capsule with ten radiopaque markers to be taken at breakfast. On day 4, a single abdominal X-ray image (posterior-anterior) will be taken. The colonic passage time in hours will be calculated according to the following formula: colonic passage time = the number of markers in the colon × 2.4 [11]. The number of each radiopaque marker in the stomach, left colon, right colon, and rectosigmoid will be recorded in a 3 × 4 table. Location of the organs on the X-ray film will be done based on the gaseous outlines of the colon. If the organs are not discernible, the method described by Metcalf et al. [11] will be applied. Markers located to the right of the vertebral spinous processes above a line from the fifth lumbar vertebrae to the pelvic outlet are assigned to the right colon. Markers to the left of the vertebral spinous processes and above an imaginary line from the fifth lumbar vertebrae to the anterior superior iliac crest are assigned to the left colon. Markers inferior to a line from the pelvic brim on the right and the superior iliac crest on the left are judged to be in the rectosigmoid and rectum.

The postoperative hospital stay is the number of days from surgery until discharge. Criteria for hospital discharge include stable vital signs with no febrile morbidity for at least 24 hours, passage of stool, toleration of a regular diet, and the absence of other complications. Swiss health insurance policies and social issues sometimes require longer hospitalization than medically necessary. We will address this issue by recording the medically indicated hospitalization along with the actual hospitalization.

The time point when all the following criteria are met will be used to calculate the medically indicated length of hospitalization:

- Bowel movement
- Solid food is well tolerated
- No serious pain (no opioid containing medication necessary)
- Unproblematic mobilization
- Surgical wound without signs of inflammation or wound can be treated well in an outpatient setting
- Decreasing infectious/inflammatory parameters (on day 4 after surgery CRP ≤ 135 mg/l, white blood cells count ≤ 9 × 10^9^/l) [12]

Postoperative pain will be recorded three times daily using a numeric rating scale (NRS), with scores ranging from 0 (= no pain at all) to 10 (= maximal pain) in increments of 1. Additionally, the amount, type, and time of application of analgesics will be obtained from the medical and nursing records.

Postoperative mobilization will be documented for 1 week after surgery using the following categories:

- 0: 24 h in bed
- 1: Out of bed only to go to the bathroom
- 2: Out of bed on free will

The overall fluid intake will be taken from the nursing records.

Blood pressure and pulse rate are recorded, usually three times daily, during the routine visits.

The postoperative intensive care unit stay is the number of days in the intensive care unit. A stay in the recovery room exceeding 24 h is counted as an intensive care unit stay.

#### Postoperative morbidity

The following set of predefined complications will be considered as adverse events and documented:

1. Anastomotic leakage: Communication between the intraluminal and extraluminal compartments due to a defect of the integrity of the intestinal wall at the site of the anastomosis. Clinical findings suspicious of leakage (i.e., abdominal pain, fever, elevated infectious parameters, or feculent drain content) must be confirmed by contrast-enhanced CT or during relaparoscopy or laparotomy.
2. Postoperative hemorrhage: Any drop of hemoglobin > 3 g/dl (6 h after the end of surgery), any postoperative transfusion of packed red blood cells for a falling hemoglobin value, or hemorrhage requiring re-intervention (i.e., embolization or laparotomy).
3. Intraabdominal fluid collection/abscess: Intraabdominal fluid collection detected on any imaging modality (e.g., ultrasound or CT scan) associated with abdominal discomfort/pain or elevation of infectious parameters.
4. Wound infection: Pain, redness, and swelling of the wound that requires opening of the wound or antibiotic therapy.
5. Pneumonia: Pulmonary infection with evidence of increased infection parameters (CRP > 20 mg/l and/or white blood cell count > 10 × 10^9^/l) that are unlikely to be caused by a different pathologic process and evidence of pulmonary infiltrates on chest X-ray, which require antibiotic therapy.
6. Mortality: Death due to any cause during the patient’s initial hospital stay and within 30 days after surgery (by calling the patient by phone).
7. Need for invasive re-intervention: Need for re-laparoscopy, laparotomy, or other interventions (e.g., drainage) during the patient’s initial hospital stay.

#### Caffeine, nicotine, and sleeping habits evaluation

Before surgery, patients will be asked about their caffeine and nicotine consumption and their sleeping behavior as a reference for the following endpoints.

#### Well-being

No classical quality-of-life instruments (such as the SF36 or EORTC QLQ-C30) will be administered because these usually cover periods of 14–28 days, which would be too long for this study. In addition, no reason exists to assume that the quality of life would differ between the treatment and placebo group, statistically or clinically. Furthermore, a reference value would be difficult to find, as the quality of life is certainly impaired before surgery. Thus, well-being with respect to the mental state will be measured for this study, which describes the current mood and state of the patient. The Basel Mental State Scale (Basler Befindlichkeits-Skala BBS), which consists of 16 bipolar items (a choice between two properties, such as tired/awake) will be used for this study. The instrument, which covers four domains (vitality, balance, extraversion, and vigilance), is well established, validated, and suitable for Swiss and German populations (the scale was developed in Switzerland) [13]. Patients will be asked to fill in the form on the afternoon of the second and fourth day after surgery (during treatment versus after treatment).

#### Caffeine-related side effects

Patients will receive a self-administered questionnaire concerning the potential side effects of caffeine together with the well-being questionnaire mentioned above (i.e., on days 2 and 4). No established or validated instrument could be found for this purpose. Thus, the questionnaire was adopted from Liguori et al. [14] and complemented with questions regarding sleep and the patient’s self-assessment on whether the patient actually received caffeine. In addition, the established and validated Leeds sleep-evaluation questionnaire will be administered [15–17].

#### Consumption of sleep-inducing drugs

The type and amount of sleep-inducing drugs will be taken from the medical records.

#### Satisfaction with surgery

On day 4 (or before release), patients will be asked about satisfaction with the surgery. They can rate their satisfaction on a seven-step Likert scale (extremely unsatisfied to extremely satisfied).

### Ethics

This clinical study will be conducted in accordance with the current version of the World Medical Association Declaration of Helsinki, ICH-GCP, and the currently valid local regulations. The current version of the study protocol (Version 3 from 14 August 2015) has been approved by the Ethics Committee of the Canton St. Gallen (EKSG 15-023) and by Swissmedic (Swiss Agency for Therapeutic Products, www.swissmedic.ch). Patients will be informed verbally by an investigator about the aims, the design, the risks, the patient rights, and the course of the trial. They will also receive printed patient information. Only patients who have signed the informed consent form 24 h before surgery and meet all inclusion and exclusion criteria will be included in the trial.

### Study outline

If a suitable patient is identified during the outpatient visit in preparation of surgery, he or she will be informed about the trial by a surgeon or physician. If the patient is interested in participation, the course of the trial will be explained in detail to the patient, and the investigator inquires whether all inclusion and exclusion criteria are met. If this is the case, the patient will receive a printed version of the patient information and the informed consent form. The informed consent form has to be signed and returned no later than on the day before surgery.

**Fig. 1:**
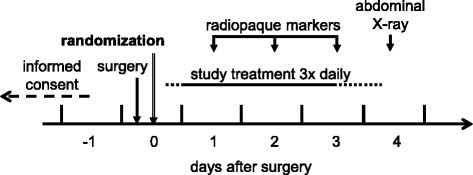
Time course of the study treatment. Study treatment starts the evening of surgery if wound dressing was applied before 1 p.m.; otherwise, in the morning of the next day. Study treatment continues until the first solid bowel movement or until ten capsules have been taken (whichever occurs first). The *dotted line* at both ends of the study treatment period indicates that the start and end of the study treatment can vary according to the above-stated rules

After surgery, the inclusion criteria will be checked again (no change in the planned course of surgery), and if all criteria are fulfilled, the patient will be randomized to receive capsules containing either 100 mg caffeine, 200 mg caffeine, or placebo (corn starch) (Fig. 1).

If the wound dressing was placed before 1 p.m., the first capsule will be given in the evening; otherwise, in the morning of the following day. Patients will take a total of ten capsules, one with each meal (three times daily). However, the medication will be stopped after the primary endpoint (normal bowel movement) has been reached. The remaining capsules will be returned to the study center. A placard will be attached to the patient bed to indicate study participation and that the patient is not allowed to consume caffeine-containing beverages or food.

In addition to the study treatment, the patients will take a capsule with radiopaque markers every morning starting with the day after surgery for 3 days. On day 4 after surgery, a patient will undergo X-ray imaging to determine the location of the markers to determine the colonic passage time.

Otherwise, the treatment will not differ from the treatment of the colectomy received outside of the study, including the treatment of an ileus after the end of the study period. In case of an ileus after the patient has taken the last study medication, the underlying medical conditions will be corrected, in particular, electrolyte or acid-base abnormalities. Patients suffering from vomiting or distention will receive a nasogastric tube. Finally, patients with an ileus will receive laxatives (magnesium salts, Laxoberon, and one enema/day) and prokinetic drugs (erythromycin 3 × 100 mg/day i.v. for 3 days, and 12–24 h later, metoclopramide 10–30 mg/day iv, together with neostigmine 0.5–1.5 mg iv).

### Randomization and blinding

Randomization will occur after surgery and inclusion/exclusion criteria have been met. Each patient is assigned with equal probability to one of three treatment arms based on a permuted block randomized list with variable block sizes of three to 15. After 60 patients (at the interim analysis) have been enrolled, the patients will be equally allocated to the three arms. The Cantonal Pharmacy of Zurich prepares the study medication based on this randomization list and packages the medication in indistinguishable containers labelled with consecutive numbers (1–180), according to the list. Medication consists of capsules of the same appearance and level of filling (corn starch was added to the capsules containing the caffeine to assure equal filling of all capsules).

For the first 20 patients, medications will be assigned in the order of surgery. After 20 patients, the second site (Rorschach) will be activated for recruiting. Batches of medications with consecutive numbers (according to demand, approximately 5–10) will be transferred to the second site. Each site will then assign the medication according to the lowest available medication number at the site.

Except for the study coordinator (who generated the randomization list) and the Cantonal Pharmacy, no one has access to the randomization list or has knowledge of the allocation sequence (the study coordinator keeps a password-encrypted and protected copy). Thus, neither the study participant nor the investigator or nursing staff have knowledge of the treatment assignment. In addition, the statistician will analyze the data while blinded to the treatment allocation (treatment arms will be coded with A, B, and C).

In case of unforeseen adverse events that require a knowledge of the treatment allocation, an emergency unblinding procedure is available: an envelope corresponding to the medication number will be added to the patient file of each study participant. This envelope contains a 12-character code (numerals and case sensitive letters) specific for each medication number. The medication number and the code can be entered in a generally accessible program, which then will reveal the treatment assignment. Any surgeon of the department can perform this procedure; however, the surgeon will be asked to give a reason for unblinding. The user name and the computer ID, as well as the time of unblinding, will be recorded, and a message will be sent to the study coordinator.

Technically, the 12-character code is a random key phrase to decode the AES encoded treatment assignment stored in the program. Thus, neither the code in the envelope nor the (encrypted) data in the program alone can be used to derive the treatment assignment. Only the medication number-specific combination of the code in the envelope together with the encrypted data in the program will allow unblinding of the given medication number.

### Statistical analysis

One interim analysis is planned to assess the primary outcome and the safety of the treatment after 60 patients have been randomized. The statistician will provide an analysis of the primary outcome and a summary of adverse events (in particular, adverse events attributable to caffeine). In this analysis, the arm assignments will not be revealed (blinded); the arms are only labeled A, B, and C. The results will be presented to the data and safety monitoring committee (DSMC). Based on these data, the trial chairperson and the DSMC will decide whether to continue the trial without modification, continue the trial with modification, or stop the trial due to safety or efficacy concerns. If in doubt, the DSMC can ask to reveal the arm assignments before making a final decision. Early stopping of the trial for efficacy at the interim analysis should only be instituted if the benefit of the treatment is shown “beyond reasonable doubt.” A nominal p-value < 0.0007 (O’Brien Fleming alpha spending, see below) provides guidance in this instance but is not binding for this decision. No formal criteria for early stopping for safety reasons will be instituted. The DSMC will make this decision based on statistical analysis, as well as on clinical judgment.

The results of the interim analysis will be confidential and strictly limited to the trial statistician and the trial chair and co-chairs, as well as the DSMC. This confidentiality should prevent any bias by the interim results on the continuation of the trial.

Statistical analysis will be performed with a current R environment (www.r-project.org). For baseline characteristics, descriptive statistics will be used as appropriate. All confirmatory analyses will primarily be performed as an intention-to-treat-analysis, assessing all patients with available data for the outcomes according to the randomization. The significance level alpha for analysis of the primary outcome will be adjusted to the exact proportion of information included in the interim analysis if the analysis is not performed at exactly 60 enrolled patients. For all other analyses, two-sided significance tests with an alpha of 0.05 will be applied.

To maintain the alpha level, an O’Brien Fleming alpha spending function will be used [18]. The formal significance level in the interim analysis after 60 enrolled patients is alpha = 0.0007. The alpha for the final analysis will be 0.0498 for the assessment of the time to first bowel movement in the main analysis.

For the final analysis, the superiority of caffeine over placebo for the time to first bowel movement (primary outcome) will be assessed irrespective of the caffeine dosage using a Mann-Whitney-U-statistic. Hence, the arms with 100 mg and 200 mg caffeine will be treated as one arm. To maintain the alpha level despite the interim analysis, the alpha for testing the hypothesis is 0.0498.

Secondary continuous outcomes will be assessed by regression analysis of the mean ranks considering the caffeine dosage. Colon transit time analysis will be performed while additionally stratified by gender, as colon transit time is significantly shorter in men. Categorical secondary outcomes will be assessed by logistic regression analysis considering the caffeine dosage. Final analysis will be done according to the intention-to-treat principle and compared to per-protocol analyses. Auxiliary nonconfirmatory analyses will be performed to assess the influence of baseline and treatment characteristics on the primary and secondary outcomes.

### Sample size calculation

Sample size calculation was performed for the main outcome, the time to first bowel movement using the R environment version 3.0, and the “gsDesign” and “samplesize” packages.

In a previous study, coffee consumption reduced the time to bowel movement after surgery from 74 ± 21 h to 60 ± 21 h (mean ± standard deviation) [10]. In that study, one cup of coffee (containing 80 to 130 mg of caffeine [19]) was administered three times a day. The published results of this study were based on a mix of open and laparoscopic procedures in patients with and without epidural analgesia. Because we have access to the raw data of this study, possible bias by the type of surgical access and epidural analgesia could be eliminated. Using the raw data (per-protocol analysis), the time to first bowel movement for patients after open colon resection with epidural analgesia was 61.5 ± 18.5 h for the 21 patients receiving coffee versus 72.5 ± 19.5 h for the 25 patients receiving the placebo. The number of patients with laparoscopic surgery was too low to derive meaningful data. The standardized effect size of caffeine was estimated to be 0.5 (a reduction of 10 h with a standard deviation of 20 h). Even if the absolute time to bowel movement after laparoscopic colectomy differs from the time after open surgery, the standardized effect size for both types of access can be assumed comparable. In addition, a standardized effect size of 0.5 is usually considered to be of clinical importance for study outcomes.

Assuming a power of 80 % with a two-sided alpha of 0.05 (hypothesis: superiority of caffeine), a total of 144 patients (N = 48 in the placebo, and N = 96 in the treatment arms) are necessary to detect a standardized effect size of 0.5 for a 1:2 ratio of placebo versus caffeine (for this analysis, the two caffeine arms (100 mg/200 mg) are pooled). Assuming a 20 % dropout rate (withdrawal rate), 180 patients are required for the study. Patients who withdraw from the study before receiving the first study treatment (caffeine, placebo, or radio-opaque markers) will be replaced by other patients. Otherwise no replacement for dropout or withdrawals will be performed. A bootstrap simulation using the raw data mentioned above confirmed the parametrically determined sample size.

### Data collection and monitoring

Patients will be hospitalized during the whole study (due to the colectomy). Thus, study participants are under steady monitoring by surgeons and regular nursing staff. Furthermore, a dedicated study nurse will collect data from the patients’ records and will ask the patients directly for the key endpoints (time to first flatus, time to bowel movement, etc.). This study nurse will also deliver and collect the questionnaires from the patients and will help them with the questionnaires if the patient so wishes.

Data will be collected with a custom-made software with extensive data-entry checks (numerical data are restricted to plausible ranges, and categorical data entry is performed via pull-down menus or option buttons). Entry forms for questionnaires have the same layout as the questionnaires themselves. Thus, correct data entry can be confirmed by simple pattern comparison. Data entry is recorded in an audit log, the data set can be restored to the state of the last five close events, and an overnight backup to a remote server is performed daily.

### Patient safety

Since patients are hospitalized during the whole study period, they are well monitored and adverse events can be quickly treated. Most people in the Western world consume caffeine-containing beverages on a regular basis. The lower dose of caffeine used in this study (100 mg) corresponds to approximately 1 cup of filtered coffee. Furthermore, numerous medications contain caffeine as an additional ingredient (e.g., painkillers); thus, one can assume that the consumption of caffeine, even at the higher dose of 3 × 200 mg is very safe for the patients. However, few data exist on the effect of caffeine on elderly (most patients presumably will be older than 50 years) [20, 21], and none, on the consumption right after abdominal surgery.

Most of the documented side effects are not very severe and are usually observed after higher dosages. Documented side effects are muscle tremor (>200 mg), sleeplessness, restlessness, headache (>200 mg), irritability, gastrointestinal complaints, tachycardia, and, in rare cases, allergic reactions. Doses of 100 mg are not recommended in the case of tachyarrhythmia and liver cirrhosis. All these side effects and restrictions have been considered in the exclusion criteria [22].

While all available knowledge suggests that the consumption of caffeine after surgery should be safe, unexpected adverse effects cannot be ruled out with certainty. In case a patient should be harmed by the trial treatment, the sponsor obtained a liability insurance to cover any damages caused by the trial participation (as required by Swiss law).

The safety and efficacy of the study treatment is being monitored by a data and safety monitoring committee consisting of three members who are independent of the study. The members are specialists in hepatology, emergency medicine, and surgery.

### Publication

The results of this trial are intended for publication in a peer-reviewed medical journal independently of the outcome of the trial. Trial participants will be provided with a German summary of the trial results or a copy of the final publication if they expressed their interest on the informed consent form to receive one or both of these documents.

## Discussion

A previous study showed that postoperative coffee consumption shortens the time to first bowel movement after colorectal resections [10]. As outlined in the Background section, it is highly unlikely that this effect is due to the physical properties (temperature, osmolality, or volume) of the coffee. Much more likely, one (or more) of the numerous phytochemicals of the coffee bean are responsible for the effect. The most obvious candidate seems to be caffeine, the most popular and probably best-researched component of the coffee. However, very little evidence exists that caffeine was responsible for the observed effect in the study by Müller et al. [10]. Rao et al. reported that the effect of caffeinated coffee on colonic function is stronger than decaffeinated coffee; however, even decaffeinated coffee had an effect on the colonic function [9]. Whether this effect was due to the low amount of caffeine still present in the decaffeinated coffee or due to another substance is unknown. Thus, we agree that our hypothesis for this trial is rather speculative. Nevertheless, the safety profile of caffeine is well documented, and the substance is part of the daily diet of most people in the Western world. In case our study hypothesis should be wrong, the risk of doing harm to the study participants is extremely low. Therefore, we think this study is ethically acceptable, particularly considering that, if the hypothesis were proven, a cost-effective and well-accepted drug with little side effects would be available to ameliorate postoperative bowel paralysis.

### Trial status

Since 1 August 2015, the trial is open for recruiting, and so far, 12 patients have been randomized (as of 7 March 2016).

## Additional file

Additional file 1: SPIRIT checklist. Checklist and page references of the SPIRIT items. (DOCX 62 kb)

## Abbreviations

AES: advanced encryption standard (current approved and standard algorithm for data encryption)
ASA: American Society of Anesthesiologists, here used as an acronym for the ASA physical status classification system
CRP: C-reactive protein
CT: computed tomography
NRS: numeric rating scale
